# National Trends and Disparities in Herpes Zoster Vaccination Among US Older Adults With Diabetes, 2008–2023

**DOI:** 10.1002/pds.70301

**Published:** 2025-12-12

**Authors:** Chun‐Tse Hung, Li‐Min Wang, Ding‐Cheng Liu, Yu‐Chien Hung

**Affiliations:** ^1^ School of Pharmacy, College of Pharmacy Taipei Medical University Taipei Taiwan; ^2^ Department of Clinical Pharmacy, College of Pharmacy University of Michigan Ann Arbor Michigan USA; ^3^ Department of Pharmacy Taipei Medical University Hospital Taipei Taiwan; ^4^ Department of Regulatory and Quality Sciences, School of Pharmacy and Pharmaceutical Science University of Southern California Los Angeles California USA; ^5^ School of Medicine, College of Medicine National Yang Ming Chiao Tung University Taipei Taiwan

**Keywords:** diabetes, herpes zoster, immunization, National Health Interview Survey, vaccination, vaccines

## Abstract

**Purpose:**

To evaluate trends and disparities in herpes zoster vaccination among US older adults with diabetes.

**Methods:**

Data from the 2008 to 2023 National Health Interview Survey were used. Joinpoint regression analysis was performed to analyze trends in herpes zoster vaccination. A multivariable logistic regression model was used to identify factors associated with herpes zoster vaccination.

**Results:**

A total of 42 377 participants with diabetes were included, representing approximately 18 million US older adults with diabetes. From 2008 to 2023, the prevalence of herpes zoster vaccination increased nearly tenfold, from 4.2% in 2008 to 42.2% in 2023 (average annual percent change = 14.09, *p* < 0.01), with similar overall trends observed in adults without diabetes (*p* = 0.08). Upward trends were also observed across age groups and diabetes types. Several factors, including age, race/ethnicity, region, educational level, health insurance, income, perceived health status, flu and pneumococcal vaccination, comorbid atherosclerotic cardiovascular disease and cancer, were associated with herpes zoster vaccination.

**Conclusion:**

Herpes zoster vaccine coverage has surged among US older adults with diabetes over the past 16 years. However, disparities in vaccination remain, underscoring the need for targeted policies and interventions to improve coverage.

## Key Points

- From 2008 to 2023, the proportion of US adults aged ≥ 50 years with diabetes who received the shingles vaccine increased from 4.2% to 42.2%, showing a significant upward trend.
- Vaccination rates were lower among Hispanic and Black adults, those with lower income or education, and those with heart disease, indicating inequities in vaccine access.
- Older age, higher income, higher education, having health insurance, receiving flu or pneumococcal vaccines, and those with cancer were linked to higher odds of getting the shingles vaccine.
- Adults with type 2 diabetes had higher shingles vaccination rates (34.9%) than those with type 1 diabetes (28.9%) from 2019 to 2023.
- About 8.7% of adults with diabetes received the recommended two doses of the Shingrix vaccine from 2019 to 2023, with 78.1% of those who started the series completing it.

## Plain Language Summary

Shingles, or herpes zoster, is a painful rash caused by the reactivation of the chickenpox virus. It affects about one in three people in their lifetime, especially older adults and those with diabetes, who are at higher risk due to weakened immune systems. This study looked at how many US adults aged 50 and older with diabetes got the shingles vaccine between 2008 and 2023, using data from a national health survey. We found that vaccination rates increased significantly over this period, from 4.2% in 2008 to 42.2% in 2023. However, vaccination rates varied by age, race, income, education, and other factors. For example, older adults, those with higher incomes, and those who got flu or pneumococcal vaccines were more likely to be vaccinated. People with type 2 diabetes had higher vaccination rates than those with type 1 diabetes. Despite the progress, some groups, like Hispanic and Black adults, or those with heart disease, were less likely to get vaccinated. These findings show the need for policies and programs to make shingles vaccines more accessible to all adults with diabetes, especially those in underserved groups.

## 1 Introduction

Herpes zoster, or shingles, is a painful and blistering rash caused by the reactivation of latent varicella zoster virus [1]. Approximately one in three individuals will develop herpes zoster in their lifetime, leading to 1 million cases annually in the United States [1, 2, 3]. The risk of herpes zoster increases with age. The incidence rate was about 6–8/1000 person‐years at 60 years of age and 8–12/1000 person‐years at 80 years of age [4], resulting in substantial morbidity among the elderly [2, 3]. Two herpes zoster vaccines have been approved in the United States [5, 6]. The first, a live attenuated vaccine called Zostavax, was licensed in 2006 for the prevention of herpes zoster in adults aged ≥ 60 years, with its indication later extended to adults aged ≥ 50 years in 2011 [5, 6]. The second vaccine, Shingrix, is a two‐dose recombinant zoster vaccine introduced in the United States in 2017 [7]. Since 2008, the US Advisory Committee on Immunization Practices (ACIP) has recommended herpes zoster vaccination for adults aged ≥ 60 years [6]. In 2017, the ACIP extended this recommendation to adults aged ≥ 50 years [7].

Diabetes is a risk factor for developing herpes zoster, likely due to impaired cell‐mediated immunity in patients with diabetes [1, 8]. An increased risk of herpes zoster has been observed in patients with both type 1 and type 2 diabetes [9, 10]. Additionally, the loss of cell‐mediated immunity appears to correlate with the duration of diabetes [11]. Given the elevated risk, promoting herpes zoster vaccination among patients with diabetes is important [8, 11]. The American Diabetes Association (ADA) includes herpes zoster vaccines among the highly recommended vaccinations for adults with diabetes [12]. However, evidence on vaccine uptake among patients with diabetes remains limited, as most previous studies have focused on vaccination coverage in the general adult population [2, 3, 13, 14] or other chronic conditions [5, 15, 16]. Furthermore, recommendations and availability for herpes zoster vaccines have changed over time [6, 7]. Therefore, a comprehensive investigation into trends and disparities in vaccination among patients with diabetes is crucial.

This study aimed to (1) evaluate trends in herpes zoster vaccination and (2) identify factors associated with herpes zoster vaccination among US older adults with diabetes.

## 2 Methods

### 2.1 Data Source

Data were obtained from the 2008 to 2023 National Health Interview Survey (NHIS). NHIS is a nationally representative household survey conducted by the National Center for Health Statistics (NCHS) [17]. NHIS is the principal source of information on the health of the US noninstitutionalized civilians [17]. NHIS employs a complex sampling design involving stratification, clustering, and weighting to represent the US population [17]. Weights account for sampling probabilities and are provided by the NCHS in NHIS data files to generate national estimates [17]. Data collection in the NHIS was approved by the NCHS Ethics Review Board [17].

### 2.2 Study Population

Participants aged ≥ 50 years were identified as older adults. Participants with diabetes were defined as those with an affirmative response to the question: “Have you ever been told by a doctor or health professional that you have diabetes or sugar diabetes?” [18, 19, 20].

### 2.3 Outcome

Herpes zoster vaccination was the study outcome. From 2008 to 2017, this was identified by the question: “Have you ever had the Zoster or Shingles vaccine, also called Zostavax?” [5, 13, 15, 16, 17]. From 2018 to 2023, the question was slightly changed to: “Have you had a vaccine for shingles?” [5, 13, 15, 16, 17]. An affirmative response to these questions was considered evidence of herpes zoster vaccination [5, 13, 15, 16, 17].

### 2.4 Covariates

Covariates encompassed three aspects: demographics, comorbidities, and diabetes‐related factors. Demographics included age (50–59, 60–69, and ≥ 70 years old), sex (male and female), race/ethnicity (non‐Hispanic White, Hispanic, Non‐Hispanic Black, and others), region (Northeast, Midwest, South, and West), educational level (less than high school, and high school and higher), health insurance (covered and not covered), family income as a percentage of the federal poverty level (FPL) (< 100%, 100% to < 200%, 200% to < 400%, and ≥ 400%), perceived health status (good and poor), and flu and pneumococcal vaccination status (yes and no). Comorbidities included asthma, chronic obstructive pulmonary disease (COPD), obesity, hypertension, atherosclerotic cardiovascular disease (ASCVD, defined as having coronary artery disease, myocardial infarction, or stroke), and cancer. These comorbidities were selected due to their common occurrence in patients with diabetes, or their associations with increased risks of herpes zoster [1, 12]. Diabetes‐related factors included years since diabetes diagnosis (< 10 and ≥ 10 years), current use of oral glucose‐lowering medications (yes and no), and insulin (yes and no).

### 2.5 Statistical Analysis

In Aim 1, the prevalence of herpes zoster vaccination among US older adults was estimated and stratified by the presence of diabetes. Survey‐weighted percentages were calculated based on NCHS analytic guidelines to obtain national estimates [21]. Taylor series linearization was used to estimate standard errors for percentages, accounting for the complex sampling design [21]. Joinpoint regression analysis was used to analyze trends in herpes zoster vaccination. This method models data using several connected linear segments [22, 23]. Each joinpoint represents the year where two segments with different slopes meet [22, 23]. Annual percentage change (APC) and average annual percentage change (AAPC) were calculated, with significant differences from zero reported [18]. The APC represents the yearly percent change in vaccination within each time segment (i.e., from the starting point to the first joinpoint, between two consecutive joinpoints, or from the last joinpoint to the ending point) [22, 23]. The AAPC was calculated as the weighted geometric mean of the APCs, with weights corresponding to the length of each segment within the specified period [22, 23]. The comparability test of coincidence was used to determine whether the two joinpoint regression functions were identical [23, 24].

In Aim 2, a Rao–Scott *χ* ^2^ test was used to evaluate differences in the prevalence of herpes zoster vaccination across all covariates among older adults with diabetes. A multivariable logistic regression model was applied to identify factors associated with herpes zoster vaccination, adjusting for all covariates and survey year. Statistical significance was assessed with a two‐tailed *α* of 0.05. Joinpoint regression analysis was conducted using the Joinpoint Regression Program by the National Cancer Institute, version 5.2.0 [23]. Other analyses were performed with SAS 9.4 (Cary, NC).

### 2.6 Sensitivity Analysis

To assess the robustness of results, a sensitivity analysis was conducted for Aim 1. Trends in herpes zoster vaccination were stratified by age groups (50–59, 60–69, and ≥ 70 years) to evaluate the influence of changes in the US ACIP recommendations regarding age [6, 7].

### 2.7 Subgroup Analysis

A subgroup analysis was conducted for participants surveyed in 2019–2023. First, trends in herpes zoster vaccination were stratified by diabetes types (types 1 and 2). Second, the prevalence and distribution of the number of Shingrix doses received by participants were estimated. Information on Shingrix was obtained via the following questions: “Have you ever had any Shingrix shots?” and “How many Shingrix shots have you ever had?” [5, 16].

## 3 Results

Table 1 shows the prevalence of herpes zoster vaccination among US older adults in 2008–2023. A total of 42 377 participants with diabetes were included, representing approximately 18 million US older adults with diabetes. The overall prevalence of herpes zoster vaccination among US older adults was 21.2%. When stratified by the presence of diabetes, the prevalence was 22.2% for older adults with diabetes and 21.0% for those without (*p* < 0.01).

**TABLE 1:** Prevalence of herpes zoster vaccination among US older adults by the presence of diabetes in 2008–2023.

	*n*	*N*	%	SE	*p* ^a^
Total population					
Adults aged 50 and over	248 477	108 464 748	—	—	—
With diabetes	42 377	18 048 980	—	—	—
Without diabetes	206 100	90 415 768	—	—	—
Herpes zoster vaccination					
Adults aged 50 and over	57 706	23 044 057	21.2	0.2	< 0.01
With diabetes	10 010	4 013 941	22.2	0.3	
Without diabetes	47 696	19 030 116	21.0	0.2	

Abbreviations: %, percentage of the weighted population; *n*, number of the unweighted population; *N*, number of the weighted population; SE, standard error of the percentage.

^a^ For the comparison of percentages among adults aged 50 and over with and without diabetes.

**FIGURE 1:**
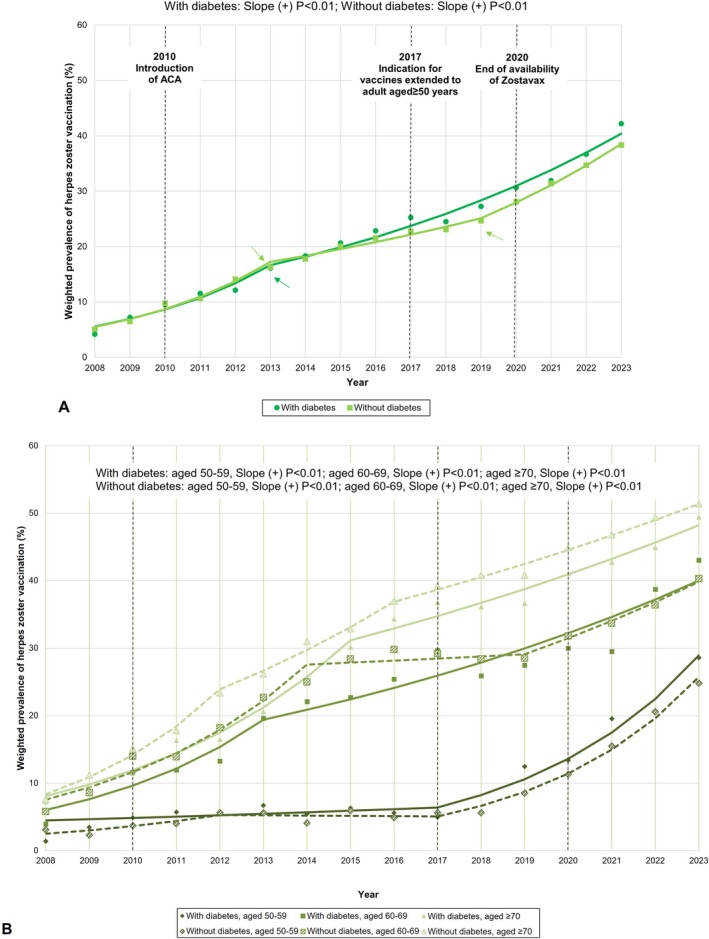
Trends in herpes zoster vaccination among US older adults. The model‐based trends (lines) and observed prevalence estimates (dots) are depicted. The *p* value reported in the figure referred to the trend from 2008 to 2023. Arrows indicate the joinpoints identified from the joinpoint regression analysis. (A) Overall, stratified by the presence of diabetes. (B) Stratified by age groups and the presence of diabetes. ACA, Affordable Care Act.

Table S1 presents annual prevalence estimates of herpes zoster vaccination among US older adults. Figure 1A illustrates the overall trend in vaccination from 2008 to 2023. Among older adults with diabetes, the prevalence of herpes zoster vaccination increased nearly tenfold—from 4.2% in 2008 to 42.2% in 2023 (AAPC = 14.09, *p* < 0.01), with a joinpoint identified in 2013 (Table 2). This upward trend was consistent across age groups: 50–59 (AAPC = 13.24, *p* < 0.01), 60–69 (AAPC = 13.45, *p* < 0.01), and ≥ 70 years (AAPC = 12.64, *p* < 0.01) (Table 2 and Figure 1B). Among adults aged 50–59, the trend was stable from 2008 to 2017 (APC = 4.05, *p* = 0.34), followed by a significant increase from 2017 to 2023 (APC = 28.56, *p* < 0.01). Similar patterns were observed among those aged 60–69 and ≥ 70, with joinpoints in 2013 and 2015, respectively. For older adults without diabetes, increasing trends in vaccination were observed for the overall (AAPC = 13.86, *p* < 0.01) and age groups (aged 50–59, AAPC = 16.84, *p* < 0.01; aged 60–69, AAPC = 11.72, *p* < 0.01; aged ≥ 70, AAPC = 12.85, *p* < 0.01) (Figure 1B). When comparing vaccination trends between older adults with and without diabetes, overall trends were similar (*p* = 0.08) (Figure 1A). Age‐stratified analyses showed similar trends between groups aged 50–59 (*p* = 0.18) and 60–69 (*p* = 0.06). However, among adults aged ≥ 70, vaccination rates were consistently higher in those without diabetes compared to those with diabetes (*p <* 0.01) (Figure 1B).

**TABLE 2:** Trends in the prevalence of herpes zoster vaccination among US older adults with diabetes in 2008–2023: Results from the joinpoint regression analysis.^a^

	Segment 1			Segment 2			Full range	
	Period	APC (95% CI)	*p*	Period	APC (95% CI)	*p*	AAPC (95% CI)	*p*
Overall	2008–2013	24.35 (15.12, 80.11)	< 0.01	2013–2023	9.28 (6.28, 11.15)	< 0.01	14.09 (12.09, 20.62)	< 0.01
Age (years)								
50–59	2008–2017	4.05 (−4.63, 13.53)	0.34	2017–2023	28.56 (19.80, 37.97)	< 0.01	13.24 (7.40, 19.39)	< 0.01
60–69	2008–2013	26.35 (4.98, 52.06)	0.02	2013–2023	7.50 (5.05, 10.01)	< 0.01	13.45 (7.20, 20.06)	< 0.01
≥ 70	2008–2015	21.26 (18.23, 26.22)	< 0.01	2015–2023	5.60 (4.49, 6.72)	< 0.01	12.64 (11.79, 13.92)	< 0.01

Abbreviations: AAPC, average annual percent change; APC, annual percent change; CI, confidence interval.

^a^ The annual percent change reflects the yearly percent change in vaccination within each time segment, while the average annual percent change is the weighted geometric mean of annual percent changes across all segments. Joinpoint regression identifies points (“joinpoints”) where trends change significantly.

Table 3 presents characteristics and factors associated with herpes zoster vaccination among US older adults with diabetes in 2008–2023. Vaccination rates were highest among patients aged ≥ 70 years (30.8%), followed by those aged 60–69 (23.7%) and 50–59 (9.3%), with adjusted odds ratios (ORs) of 2.96 (95% confidence interval [CI]: 2.65–3.29) and 2.40 (95% CI: 2.16–2.68), respectively, compared to the 50–59 age group. Vaccination rates were similar between males and females (22.0% vs. 22.5%, *p* = 0.40). Non‐Hispanic Whites had the highest vaccination rates (25.5%) compared to Hispanics (15.5%; OR 0.77, 95% CI: 0.69–0.87) and non‐Hispanic Blacks (15.0%; OR 0.71, 95% CI: 0.64–0.79). Factors associated with higher odds of vaccination included residing in the West (OR 1.32, 95% CI: 1.17–1.50), having high school and higher (OR 1.28, 95% CI: 1.15–1.42), having health insurance coverage (OR 1.41, 95% CI: 1.02–1.94), having a family income of 200% to < 400% (OR 1.32, 95% CI: 1.17–1.50) or ≥ 400% of the FPL (OR 1.76, 95% CI: 1.55–1.99), perceiving good health status (OR 1.26, 95% CI: 1.13–1.41), receiving flu vaccination (OR 2.38, 95% CI: 2.17–2.61), receiving pneumococcal vaccination (OR 3.33, 95% CI: 3.04–3.64), and having cancer (OR 1.09, 95% CI: 1.01–1.18). Conversely, having ASCVD was associated with lower odds of vaccination (OR 0.81, 95% CI: 0.75–0.87). Neither diabetes duration ≥ 10 years (OR 1.04, 95% CI: 0.97–1.11) nor use of oral glucose‐lowering medications (OR 1.03, 95% CI: 0.94–1.12) or insulin (OR 0.99, 95% CI: 0.91–1.07) were associated with vaccination.

**TABLE 3:** Characteristics and factors associated with herpes zoster vaccination among US older adults with diabetes: Descriptive statistics and regression model.

Characteristics^a^	Herpes zoster vaccination								
	Yes (*N* = 4 013 941)			No (*N* = 14 035 039)			*p*	Odds ratio^c^	95% CI
	*N*	%	SE	*N*	%	SE			
Age (years)							< 0.01		
50–59	476 859	9.3	0.4	4 623 776	90.7	0.4		Reference	Reference
60–69	1 503 499	23.7	0.5	4 834 833	76.3	0.5		2.40	(2.16–2.68)
≥ 70	2 033 583	30.8	0.5	4 576 430	69.2	0.5		2.96	(2.65–3.29)
Sex							0.40		
Male	2 014 170	22.0	0.4	7 131 521	78.0	0.4		Reference	Reference
Female	1 999 771	22.5	0.4	6 903 518	77.5	0.4		1.03	(0.96–1.11)
Race/ethnicity							< 0.01		
Non‐Hispanic White	2 910 701	25.5	0.4	8 504 287	74.5	0.4		Reference	Reference
Hispanic	415 978	15.5	0.6	2 267 418	84.5	0.6		0.77	(0.69–0.87)
Non‐Hispanic Black	404 775	15.0	0.6	2 298 898	85.0	0.6		0.71	(0.64–0.79)
Others	282 487	22.7	1.1	964 436	77.3	1.1		0.89	(0.78–1.03)
Region							< 0.01		
Northeast	665 481	21.9	0.7	2 373 740	78.1	0.7		Reference	Reference
Midwest	953 604	24.0	0.6	3 024 319	76.0	0.6		1.11	(0.99–1.25)
South	1 482 671	20.1	0.4	5 883 144	79.9	0.4		0.99	(0.88–1.10)
West	912 185	24.9	0.7	2 753 837	75.1	0.7		1.32	(1.17–1.50)
Educational level							< 0.01		
Less than high school	484 825	16.3	0.6	2 494 461	83.7	0.6		Reference	Reference
High school and higher	3 518 695	23.4	0.3	11 495 993	76.6	0.3		1.28	(1.15–1.42)
Health insurance							< 0.01		
Not covered	55 391	6.1	0.8	858 259	93.9	0.8		Reference	Reference
Covered	3 954 848	23.1	0.3	13 149 065	76.9	0.3		1.41	(1.02–1.94)
Family income							< 0.01		
FPL < 100%	330 613	14.1	0.6	2 016 334	85.9	0.6		Reference	Reference
FPL 100% to < 200%	721 915	18.4	0.5	3 192 000	81.6	0.5		1.00	(0.88–1.13)
FPL 200% to < 400%	1 271 160	24.0	0.5	4 027 238	76.0	0.5		1.32	(1.17–1.50)
FPL ≥ 400%	1 474 122	28.9	0.5	3 623 090	71.1	0.5		1.76	(1.55–1.99)
Perceived health status							< 0.01		
Poor	385 238	17.1	0.6	1 862 974	82.9	0.6		Reference	Reference
Good	3 625 235	23.0	0.3	12 166 176	77.0	0.3		1.26	(1.13–1.41)
Flu vaccination							< 0.01		
No	580 559	9.3	0.3	5 689 885	90.7	0.3		Reference	Reference
Yes	3 433 382	29.1	0.4	8 345 154	70.9	0.4		2.38	(2.17–2.61)
Pneumococcal vaccination							< 0.01		
No	748 061	9.2	0.3	7 363 199	90.8	0.3		Reference	Reference
Yes	3 265 880	32.9	0.5	6 671 840	67.1	0.5		3.33	(3.04–3.64)
Comorbidities									
Asthma	462 558	22.6	0.8	1 585 668	77.4	0.8	0.63	1.02	(0.91–1.16)
COPD	561 440	23.2	0.7	1 862 503	76.8	0.7	0.13	0.97	(0.88–1.07)
Obesity	2 084 233	21.7	0.4	7 520 682	78.3	0.4	0.02	1.03	(0.96–1.11)
Hypertension	3 156 198	22.8	0.3	10 709 933	77.2	0.3	< 0.01	1.01	(0.93–1.10)
ASCVD^b^	1 157 306	22.4	0.5	4 013 039	77.6	0.5	0.70	0.81	(0.75–0.87)
Cancer	1 009 351	29.5	0.6	2 408 223	70.5	0.6	< 0.01	1.09	(1.01–1.18)
Years since diabetes diagnosis							< 0.01		
< 10	1 436 672	18.9	0.4	6 173 205	81.1	0.4		Reference	Reference
≥ 10	2 472 820	24.8	0.4	7 483 515	75.2	0.4		1.04	(0.97–1.11)
Currently taking oral glucose‐lowering medications							0.73		
No	973 994	22.1	0.5	3 435 427	77.9	0.5		Reference	Reference
Yes	3 039 946	22.3	0.3	10 599 612	77.7	0.3		1.03	(0.94–1.12)
Currently taking insulin							0.38		
No	2 817 726	22.1	0.3	9 935 771	77.9	0.3		Reference	Reference
Yes	1 196 214	22.6	0.5	4 099 268	77.4	0.5		0.99	(0.91–1.07)

Abbreviations: %, percentage of the weighted population; ASCVD, atherosclerotic cardiovascular disease; CI, confidence interval; COPD, chronic obstructive pulmonary disease; FPL, federal poverty level; *N*, number of the weighted population; SE, standard error of the percentage.

^a^ Missing data due to the nonresponse rate (participants who answered “refused,” “not ascertained,” or “don't know”) was low in our study, and we did not attempt to implement an imputation for these respondents.

^b^ Atherosclerotic cardiovascular disease was defined by having coronary artery disease, myocardial infarction, or stroke.

^c^ Results from the multivariable logistic regression model, adjusting for covariates and survey years.

**FIGURE 2:**
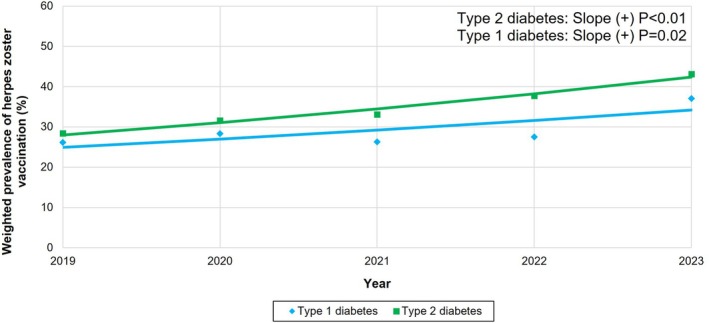
Trends in herpes zoster vaccination by types of diabetes among US older adults with diabetes in 2019–2023. The model‐based trend (line) and observed prevalence estimates (dots) are depicted. *p* value reported in the figure refers to the trend from 2019 to 2023.

Table S2 and Figure 2 present trends in herpes zoster vaccination by diabetes types. Among these patients, 16 800 816 had type 2 diabetes, accounting for 92.4% of older adults with diabetes in 2019–2023. Upward trends in vaccination were observed in patients with both type 1 and type 2 diabetes. Notably, patients with type 2 diabetes had higher vaccination rates than those with type 1 diabetes (34.9% vs. 28.9%, *p* < 0.01).

Table S3 presents estimates of Shingrix vaccination. In 2019–2023, 8.7% of older adults with diabetes received two doses of Shingrix, while 2.4% received one dose. Among those who had ever received Shingrix, 78.1% completed the two‐dose series. Higher proportions of two‐dose vaccination were also observed across all age groups.

## 4 Discussion

To our knowledge, this is the first study to evaluate trends and disparities in herpes zoster vaccination among older adults with diabetes. Over the past 16 years, there has been a rising trend in herpes zoster vaccination, both overall and across all age groups. While overall vaccination trends were similar between those with and without diabetes, notable differences emerged in the ≥ 70 age group, where adults without diabetes consistently exhibited higher vaccination rates. Upward trends were also observed by diabetes types. Disparities in vaccination were found across several factors, including age, race/ethnicity, region, educational level, health insurance, income, perceived health status, flu and pneumococcal vaccination, comorbid ASCVD and cancer.

Our findings regarding trends in herpes zoster vaccination are consistent with prior research on preventive care among patients with diabetes [25]. A US cross‐sectional study demonstrated an increasing trend in flu vaccination among adults with diabetes from 2008 to 2019 [25]. We extended this knowledge to herpes zoster vaccination, another guideline‐recommended preventive measure for patients with diabetes [12]. Additionally, previous studies have also found increasing trends in herpes zoster vaccination among the overall older adult population and those with chronic respiratory diseases or cancer [5, 13, 15, 16]. This study contributes further evidence on vaccine coverage in another at‐risk population for herpes zoster [1]. Notably, older adults with diabetes had higher vaccine coverage than those with COPD and lower vaccine coverage than those with asthma and cancer in the United States [5, 15, 16]

There are several explanations for the increasing trend in herpes zoster vaccination among patients with diabetes. The implantation of the Affordable Care Act in 2010 could have increased health insurance coverage [26], thereby improving access to healthcare and vaccine uptake among patients with diabetes. Moreover, in 2017, the US ACIP expanded the indication for herpes zoster vaccines to adults aged ≥ 50 years [7], and in the same year, the recombinant zoster vaccine, Shingrix, was introduced in the United States [5, 7]. These changes likely influenced vaccination patterns in patients with diabetes. Furthermore, the ADA included herpes zoster vaccines as important vaccinations for adults with diabetes in its clinical guidelines starting in 2018 and has recognized them as highly recommended since 2021 [27, 28]. Therefore, physicians might be more inclined to encourage patients to receive vaccines as part of comprehensive diabetes management. Despite the discontinuation of Zostavax in the United States in 2020 [29], the increasing trend in vaccination persisted, possibly due to differences in contraindications between the two vaccines [6, 7]. While Zostavax is contraindicated for immunocompromised patients and pregnant women, Shingrix is only contraindicated for individuals allergic to its components [6, 7]. Consequently, herpes zoster vaccination continued to rise, even with only one vaccine available in the United States. Finally, rising vaccination trends were also observed across all age groups. Vaccination rates among adults with diabetes aged 50–59 years have soared since 2017, further reflecting changes in the US ACIP recommendations [7].

The similarity in overall vaccination trends between adults with and without diabetes suggests that broader systemic factors, such as expanded insurance coverage and evolving clinical guidelines, have driven uptake across both groups. However, the consistently higher vaccination rates among adults aged ≥ 70 without diabetes, compared to their counterparts with diabetes, might reflect barriers specific to older adults with diabetes. These could include competing health priorities, lower engagement in preventive care, or differences in providers' recommendations [30, 31]. Further research is warranted to investigate these disparities and to inform targeted interventions aimed at improving vaccination coverage in this high‐risk population.

Prior research has suggested that patterns of healthcare utilization might differ between patients with type 1 and type 2 diabetes [19]. This was confirmed in our study, which showed that patients with type 2 diabetes had higher herpes zoster vaccination rates than those with type 1 diabetes. Patients with type 1 diabetes might have an increased risk of developing herpes zoster compared to those with type 2 diabetes [32]. Therefore, preventive strategies, particularly for patients with type 1 diabetes, could be considered given their elevated risk.

The prevalence of two‐dose Shingrix vaccination among US adults aged ≥ 50 years with diabetes was 8.7%, comparable to that of the general adults aged ≥ 50 years [14]. We also found that most patients had completed the two‐dose series of Shingrix. A cohort study with adults aged > 65 years demonstrated that the two‐dose series Shingrix provided better protection against herpes zoster than a single dose, with vaccine effectiveness rates of 70.1% and 56.9%, respectively [33]. Additionally, the US ACIP recommended administering Shingrix for two doses, given 2–6 months apart [7]. Therefore, encouraging older adults with diabetes to get fully vaccinated could enhance their protection against herpes zoster.

Consistent with prior research, older age was associated with increased odds of receiving herpes zoster vaccination [3, 5, 13, 15, 16]. This likely reflects the recommendations from the US ACIP regarding age from prior years [6, 7]. Moreover, individuals aged ≥ 65 years might have their healthcare costs covered by Medicare Part D, which could increase their willingness to be vaccinated [7]. Health insurance coverage and income were also associated with vaccination, indicating that cost‐related concerns could be a predictor of herpes zoster vaccination [18]. Therefore, ongoing efforts to mitigate financial barriers are essential to increase vaccine coverage among patients with diabetes. We found that patients with diabetes who had higher educational levels exhibited increased vaccination rates, consistent with previous studies [3, 5, 13, 15, 16]. Educational level is related to health literacy [34]. With higher levels of health literacy, patients might have a better understanding of the risk of herpes zoster, which could increase their willingness to receive vaccines. Patients with diabetes who were non‐Hispanic Black or Hispanic had lower herpes zoster vaccination coverage, possibly due to limited access to preventive care [35]. Hence, supportive policies are needed to reduce disparities in these underserved populations with diabetes.

Increased odds of receiving herpes zoster vaccine were found in patients who had flu or pneumococcal vaccinations, which was in line with previous studies [2, 5, 15]. These patients might represent a group with increased awareness of preventive care, leading to a higher vaccine coverage. Patients with diabetes who had a comorbidity of ASCVD were associated with lower odds of herpes zoster vaccination. Given that herpes zoster is associated with a higher long‐term risk of major cardiovascular events, such as stroke or coronary heart disease [36], this finding highlights the need to increase vaccination among these patients. Finally, patients with a diabetes diagnosis of ≥ 10 years had higher vaccination rates. Long duration of diabetes has been linked to an impaired immune response [11, 37]. Given the heightened risk of infection, these patients might be more likely to seek vaccination to prevent herpes zoster. Since herpes zoster could contribute to worsening glycemic control [8], proactively recommending to patients with a long duration of diabetes can be considered.

From a clinical perspective, several strategies can be considered to promote vaccination among patients with diabetes. While physicians play a pivotal role in influencing patients' vaccination willingness [38], expanding the dissemination of information and vaccine delivery sites is also important. For instance, involving pharmacists in community pharmacies could provide valuable opportunities for vaccination [39]. Moreover, healthcare providers can educate patients with diabetes about the pros and cons of herpes zoster vaccines and engage them in shared decision‐making. Public health campaigns could offer culturally tailored educational materials in multiple languages and collaborate with community‐based organizations to enhance health literacy and vaccine acceptance. Additionally, home‐based vaccination services or partnerships with aging‐focused organizations, such as Area Agencies on Aging, could help reduce disparities in vaccination [40]. This study also provides a basis for policymakers to reference when facilitating vaccinations for at‐risk populations. Beginning in 2023 in the United States, individuals with Medicare Part D coverage were covered for the Shingrix vaccine [41]. Future monitoring on trends and disparities in herpes zoster vaccines coverage is required to ensure equity in vaccinations.

This study has several strengths. First, the findings are highly generalizable to the US population, as robust estimates were obtained using nationally representative data. Moreover, this study was conducted with the most recent data available, spanning over 16 years, providing a comprehensive evaluation of trends and disparities in herpes zoster vaccination. There are also some limitations to this study. First, NHIS relies on self‐reporting data from participants, which can introduce recall bias. The prevalence of herpes zoster vaccination might be over‐ or underestimated. However, self‐reported vaccination status for herpes zoster in adults has shown high sensitivity (88.7% for aged 50–65 and 92.1% for aged > 65) and specificity (90.7% for aged 50–65 and 87.6% for aged > 65) [42]. Second, without medical records and lab data, patients with diabetes might be misclassified. Nevertheless, self‐reported diabetes has shown high validity, demonstrating a positive predictive value of 91.8% and a negative predictive value of 94.5% [43]. We therefore followed the approach used in previous studies on patients with diabetes using NHIS [18, 19]. Third, certain conditions that may impair immune function, such as chronic kidney disease, rheumatoid arthritis, systemic lupus erythematosus, inflammatory bowel disease, acquired immunodeficiency syndrome, and organ transplantation, were not consistently measured in the NHIS across all study years (Table S4). Information for some diseases was collected only in specific years, while others were entirely omitted across all study years (Table S4). Since this study aimed to assess trends over a continuous 16‐year period, including these inconsistently measured variables would have limited comparability. Therefore, we were unable to include these factors as covariates in our analysis. Nonetheless, we included cancer as a covariate to partially account for immunosuppressive conditions. Fourth, we were unable to establish causality due to the cross‐sectional design of NHIS. Finally, this study included participants aged 50–59 years, for whom herpes zoster vaccines were not recommended at the time they were surveyed [6]. However, a sensitivity analysis was conducted to evaluate trends by age groups, and the results were consistent with the main findings. Given that herpes zoster vaccines have been recommended for adults aged ≥ 50 years since 2017 [7], we aimed to provide evidence relevant to current clinical practice.

In conclusion, there has been an increasing trend in herpes zoster vaccination among US older adults with diabetes from 2008 to 2023. However, disparities in vaccination were found across several factors, highlighting the need for appropriate policies and interventions to improve vaccine coverage.

## Funding

The authors have nothing to report.

## Ethics Statement

This study was reviewed by the Joint Institutional Review Board at Taipei Medical University (reference number: N202402045).

## Consent

Data collection in the National Health Interview Survey was approved by the National Center for Health Statistics Ethics Review Board.

## Conflicts of Interest

The authors declare no conflicts of interest.

## Supporting information


**Table S1:** Prevalence of herpes zoster vaccination among US older adults in 2008–2023.
**Table S2:** Trends in herpes zoster vaccination by types of diabetes among US older adults with diabetes in 2019–2023.
**Table S3:** Prevalence and distribution of Shingrix vaccination among US older adults with diabetes in 2019–2023.
**Table S4:** Availability of measures for conditions associated with impaired immune function in the 2008–2023 National Health Interview Survey.

## Data Availability

The datasets analyzed during the current study are available at https://www.cdc.gov/nchs/nhis/index.html.
