# Supplementary material for: National Trends and Disparities in Herpes Zoster Vaccination Among US Older Adults With Diabetes, 2008–2023
Source: Pharmacoepidemiol Drug Saf. 2025 Dec 12;34(12):e70301. doi: 10.1002/pds.70301 (PMC12701292; doi:10.1002/pds.70301)
Supplement: Supplementary file 1 — Table S1: Prevalence of herpes zoster vaccination among US older adults in 2008–2023. Table S2: Trends in herpes zoster vaccination by types of diabetes among US older adults with diabetes in 2019–2023. Table S3: Prevalence and distribution of Shingrix vaccination among US older adults with diabetes in 2019–2023. Table S4: Availability of measures for conditions associated with impaired immune function in the 2008–2023 National Health Interview Survey. [file PDS-34-e70301-s001.docx]

**Supplementary tables**

| **Table S1.** Prevalence of herpes zoster vaccination among U.S. older adults in 2008-2023. | | | | | | | | | | | | | | | | | | | | | | | |
| --- | --- | --- | --- | --- | --- | --- | --- | --- | --- | --- | --- | --- | --- | --- | --- | --- | --- | --- | --- | --- | --- | --- | --- |
|  | **With diabetes** | | | | | | | | | | |  | **Without diabetes** | | | | | | | | | | |
| **Year** | **Overall** | |  | **Age (years)** | | | | | | | |  | **Overall** | |  | **Age (years)** | | | | | | | |
|  |  |  |  | **50-59** | |  | **60-69** | |  | **≥70** | |  |  |  |  | **50-59** | |  | **60-69** | |  | **≥70** | |
|  | % ^a^ | SE ^b^ |  | % ^a^ | SE ^b^ |  | % ^a^ | SE ^b^ |  | % ^a^ | SE ^b^ |  | % ^a^ | SE ^b^ |  | % ^a^ | SE ^b^ |  | % ^a^ | SE ^b^ |  | % ^a^ | SE ^b^ |
| 2008 | 4.2 | 0.6 |  | 1.4 | 0.7 |  | 3.9 | 1.0 |  | 7.3 | 1.4 |  | 5.1 | 0.3 |  | 3.1 | 0.4 |  | 5.8 | 0.6 |  | 7.6 | 0.7 |
| 2009 | 7.2 | 0.8 |  | 3.5 | 1.2 |  | 8.5 | 1.7 |  | 9.8 | 1.1 |  | 6.5 | 0.3 |  | 2.3 | 0.3 |  | 8.6 | 0.7 |  | 11.2 | 0.7 |
| 2010 | 9.5 | 0.8 |  | 4.9 | 1.0 |  | 11.6 | 1.5 |  | 11.5 | 1.2 |  | 9.8 | 0.4 |  | 3.7 | 0.4 |  | 14.0 | 0.8 |  | 15.2 | 0.9 |
| 2011 | 11.6 | 0.8 |  | 5.7 | 1.1 |  | 11.9 | 1.3 |  | 16.4 | 1.5 |  | 10.6 | 0.4 |  | 4.0 | 0.4 |  | 13.9 | 0.7 |  | 17.8 | 0.8 |
| 2012 | 12.1 | 0.8 |  | 5.7 | 1.0 |  | 13.3 | 1.5 |  | 16.5 | 1.4 |  | 14.1 | 0.4 |  | 5.6 | 0.4 |  | 18.2 | 0.8 |  | 23.4 | 0.9 |
| 2013 | 16.1 | 0.9 |  | 6.7 | 1.2 |  | 19.6 | 1.8 |  | 20.6 | 1.7 |  | 16.3 | 0.5 |  | 5.6 | 0.5 |  | 22.7 | 0.9 |  | 26.2 | 1.1 |
| 2014 | 18.3 | 0.9 |  | 5.5 | 1.0 |  | 22.1 | 1.8 |  | 25.7 | 1.7 |  | 17.8 | 0.4 |  | 4.1 | 0.4 |  | 25.0 | 0.9 |  | 31.0 | 0.9 |
| 2015 | 20.6 | 1.1 |  | 6.3 | 1.6 |  | 22.7 | 1.7 |  | 30.2 | 1.8 |  | 20.0 | 0.6 |  | 5.9 | 0.5 |  | 28.4 | 1.1 |  | 32.9 | 1.0 |
| 2016 | 22.9 | 1.0 |  | 5.6 | 1.1 |  | 25.4 | 1.8 |  | 34.3 | 1.8 |  | 21.6 | 0.5 |  | 4.9 | 0.4 |  | 29.8 | 1.0 |  | 37.0 | 1.0 |
| 2017 | 25.2 | 1.1 |  | 5.0 | 1.0 |  | 29.7 | 2.0 |  | 36.8 | 1.9 |  | 22.8 | 0.6 |  | 5.6 | 0.5 |  | 29.2 | 0.9 |  | 39.2 | 1.0 |
| 2018 | 24.5 | 1.1 |  | 5.8 | 1.2 |  | 25.9 | 1.8 |  | 36.2 | 1.8 |  | 23.1 | 0.5 |  | 5.6 | 0.5 |  | 28.4 | 0.9 |  | 40.8 | 1.1 |
| 2019 | 27.2 | 1.0 |  | 12.5 | 1.5 |  | 27.5 | 1.7 |  | 36.7 | 1.6 |  | 24.7 | 0.4 |  | 8.5 | 0.5 |  | 28.5 | 0.8 |  | 40.8 | 0.8 |
| 2020 | 30.7 | 1.0 |  | 13.4 | 1.9 |  | 30.0 | 1.7 |  | 41.1 | 1.7 |  | 28.1 | 0.5 |  | 11.3 | 0.6 |  | 31.8 | 0.8 |  | 44.9 | 0.9 |
| 2021 | 31.9 | 1.1 |  | 19.5 | 1.9 |  | 29.5 | 1.7 |  | 42.8 | 1.7 |  | 31.4 | 0.5 |  | 15.5 | 0.6 |  | 33.7 | 0.8 |  | 46.8 | 0.9 |
| 2022 | 36.7 | 1.1 |  | 20.7 | 2.0 |  | 38.7 | 1.8 |  | 44.9 | 1.7 |  | 34.7 | 0.6 |  | 20.6 | 0.8 |  | 36.4 | 0.9 |  | 49.4 | 0.9 |
| 2023 | 42.2 | 1.1 |  | 28.6 | 2.1 |  | 43.1 | 1.8 |  | 49.4 | 1.6 |  | 38.4 | 0.6 |  | 24.8 | 0.8 |  | 40.3 | 1.0 |  | 51.4 | 0.8 |
| ^a^ %: The percentage of the weighted population. | | | | | | | | | | | | | | | | | | | | | | | |
| ^b^ SE: The standard error of the percentage. | | | | | | | | | | | | | | | | | | | | | | | |

| **Table S2.** Trends in herpes zoster vaccination by types of diabetes among U.S. older adults with diabetes in 2019-2023. | | | | | | | | | | | | | | | |
| --- | --- | --- | --- | --- | --- | --- | --- | --- | --- | --- | --- | --- | --- | --- | --- |
| Year | 2019 | | 2020 | | 2021 | | 2022 | | 2023 | | Overall (2019-2023) | |  |  |  |
| Types of diabetes | %^b^ | SE^c^ | %^b^ | SE^c^ | %^b^ | SE^c^ | %^b^ | SE^c^ | %^b^ | SE^c^ | %^b^ | SE^c^ |  | AAPC (95% CI) | P value |
| Type 1 (N^a^=1,383,286) | 26.1 | 3.5 | 28.3 | 4.0 | 26.3 | 3.7 | 27.5 | 3.7 | 37.0 | 3.8 | 28.9 | 1.7 |  | 8.19 (1.12, 16.98) | 0.02 |
| Type 2 (N^a^=16,800,816) | 28.4 | 1.1 | 31.6 | 1.2 | 33.1 | 1.2 | 37.7 | 1.2 | 43.1 | 1.2 | 34.9 | 0.6 |  | 10.91 (9.17, 12.95) | <0.01 |
| ^a^ N: The number of the weighted population. | | | | | | | | | | | | | | | |
| ^b^ %: The percentage of the weighted population. | | | | | | | | | | | | | | | |
| ^c^ SE: The standard error of the percentage. | | | | | | | | | | | | | | | |
| Abbreviations: AAPC, average annual percent change. | | | | | | | | | | | | | | | |

| **Table S3.** Prevalence and distribution of Shingrix vaccination among U.S. older adults with diabetes in 2019-2023. | | | | | | | | | | | | | | | |
| --- | --- | --- | --- | --- | --- | --- | --- | --- | --- | --- | --- | --- | --- | --- | --- |
|  | Overall | | |  | Age (years) | | | | | | | | | | |
|  |  |  |  |  | 50-59 | | |  | 60-69 | | |  | ≥70 | | |
|  | Proportion (%)^a^ | Prevalence (%) | SE^b^ |  | Proportion (%)^a^ | Prevalence (%) | SE^b^ |  | Proportion (%)^a^ | Prevalence (%) | SE^b^ |  | Proportion (%)^a^ | Prevalence (%) | SE^b^ |
| Ever having Shingrix vaccination | 100.0 | 11.2 | 0.4 |  | 100.0 | 6.7 | 0.6 |  | 100.0 | 11.5 | 0.6 |  | 100.0 | 14.0 | 0.6 |
| 1 dose | 21.1 | 2.4 | 0.2 |  | 26.7 | 1.8 | 0.3 |  | 18.6 | 2.1 | 0.2 |  | 21.1 | 3.0 | 0.3 |
| 2 doses | 78.1 | 8.7 | 0.3 |  | 73.3 | 4.9 | 0.5 |  | 80.6 | 9.3 | 0.5 |  | 77.9 | 10.9 | 0.5 |
| Unknown | 0.8 | 0.1 | 0.0 |  | 0 | 0 | 0 |  | 0.8 | 0.1 | 0.1 |  | 1.0 | 0.1 | 0.1 |
| ^a^ The proportion is calculated by dividing the number of people who have received that number of Shingrix by the total number of people who have ever received Shingrix. | | | | | | | | | | | | | | | |
| ^b^ SE: The standard error of the prevalence. | | | | | | | | | | | | | | | |

| **Table S4.** Availability of measures for conditions associated with impaired immune function in the 2008-2023 National Health Interview Survey. | | | | |
| --- | --- | --- | --- | --- |
|  | Years available | Notes |  |  |
| Chronic kidney disease | 2008-2018 | This condition was collected from 2008 to 2018. Participants were asked whether they had weak or failing kidneys. | | |
| Rheumatoid arthritis | – | There are no specific questions for rheumatoid arthritis. Only overall arthritis or joint pain were collected. | | |
| Systemic lupus erythematosus | – | This condition is not available across all study years. |  |  |
| Inflammatory bowel disease | 2023 | This condition was only collected in 2023. |  |  |
| Acquired immunodeficiency syndrome | – | This condition is not available across all study years. |  |  |
| Organ transplantation | – | This condition is not available across all study years. |  |  |
